# A Novel Bayesian DNA Motif Comparison Method for Clustering and Retrieval

**DOI:** 10.1371/journal.pcbi.1000010

**Published:** 2008-02-29

**Authors:** Naomi Habib, Tommy Kaplan, Hanah Margalit, Nir Friedman

**Affiliations:** 1 School of Computer Science and Engineering, The Hebrew University, Jerusalem, Israel; 2 Department of Molecular Genetics and Biotechnology, Faculty of Medicine, The Hebrew University, Jerusalem, Israel; Massachusetts Institute of Technology, United States of America

## Abstract

Characterizing the DNA-binding specificities of transcription factors is a key problem in computational biology that has been addressed by multiple algorithms. These usually take as input sequences that are putatively bound by the same factor and output one or more DNA motifs. A common practice is to apply several such algorithms simultaneously to improve coverage at the price of redundancy. In interpreting such results, two tasks are crucial: *clustering* of redundant motifs, and attributing the motifs to transcription factors by *retrieval* of similar motifs from previously characterized motif libraries. Both tasks inherently involve motif comparison. Here we present a novel method for comparing and merging motifs, based on Bayesian probabilistic principles. This method takes into account both the similarity in positional nucleotide distributions of the two motifs and their dissimilarity to the background distribution. We demonstrate the use of the new comparison method as a basis for motif clustering and retrieval procedures, and compare it to several commonly used alternatives. Our results show that the new method outperforms other available methods in accuracy and sensitivity. We incorporated the resulting motif clustering and retrieval procedures in a large-scale automated pipeline for analyzing DNA motifs. This pipeline integrates the results of various DNA motif discovery algorithms and automatically merges redundant motifs from multiple training sets into a coherent annotated library of motifs. Application of this pipeline to recent genome-wide transcription factor location data in *S. cerevisiae* successfully identified DNA motifs in a manner that is as good as semi-automated analysis reported in the literature. Moreover, we show how this analysis elucidates the mechanisms of condition-specific preferences of transcription factors.

## Author Summary

Regulation of gene expression plays a central role in the activity of living cells and in their response to internal (e.g., cell division) or external (e.g., stress) stimuli. Key players in determining gene-specific regulation are transcription factors that bind sequence-specific sites on the DNA, modulating the expression of nearby genes. To understand the regulatory program of the cell, we need to identify these transcription factors, when they act, and on which genes. Transcription regulatory maps can be assembled by computational analysis of experimental data, by discovering the DNA recognition sequences (motifs) of transcription factors and their occurrences along the genome. Such an analysis usually results in a large number of overlapping motifs. To reconstruct regulatory maps, it is crucial to combine similar motifs and to relate them to transcription factors. To this end we developed an accurate fully-automated method, termed BLiC, based upon an improved similarity measure for comparing DNA motifs. By applying it to genome-wide data in yeast, we identified the DNA motifs of transcription factors and their putative target genes. Finally, we analyze motifs of transcription factor that alter their target genes under different conditions, and show how cells adjust their regulatory program in response to environmental changes.

## Introduction

**Figure 1:**
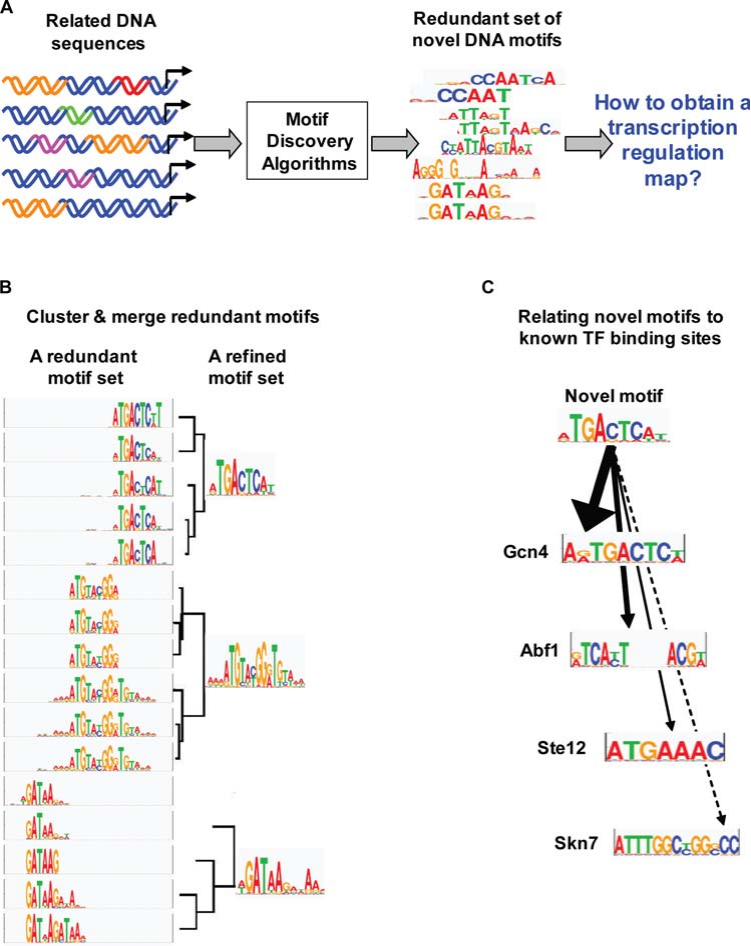
Overview of the challenges in DNA motif analysis. (A) Identifying DNA binding motifs: Applying motif discovery algorithms to a group of related DNA sequences leads to the identification of putative transcription factor DNA binding sites. These algorithms output a set of DNA motifs, which are frequently redundant. To infer the correct transcription regulation map from the discovered motif set, it is crucial to reduce this redundancy and to relate the discovered motifs to known ones. (B) Reducing redundancy by clustering and merging motifs: A redundant set of DNA motifs can be reduced by clustering the motifs into groups of related ones and merging the motifs within each cluster. In this example, a redundant set of 16 DNA motifs (a partial output of several motif search algorithms) is clustered and merged to a final set consisting of three DNA motifs. (C) Relating motifs to known factors: The transcription factors that bind the newly discovered DNA motifs can be revealed based on similarities to previously defined motifs. In this example, comparison of a newly discovered motif to four known motifs reveals high similarity to the Gcn4 binding motif. From this comparison the transcription factor that binds the motif is identified with high probability.

Transcription initiation is modulated by transcription factors that recognize sequence-specific binding sites in regulatory regions. The organization of binding sites around a gene specifies which factors can bind to it and where, and consequently determines to what extent the gene is transcribed under different conditions. To understand this regulatory mechanism, one must specify the DNA binding preferences of transcription factors. These preferences are usually characterized by a motif that summarizes the commonalities among the binding sites of a transcription factor [1]. Multiple tools were developed for finding motifs (e.g., [2]–[5]), however there are several problems in interpreting their output. Typically these algorithms output multiple results which require filtering and scoring. Moreover, different motif discovery methods have complementary successes, and therefore it is beneficial to apply multiple methods simultaneously and collate their results [6]. In addition, the motif discovery algorithms frequently produce a redundant output and the transcription factor that binds each motif is usually unknown. As similar motifs may represent binding sites of the same factor, eliminating this redundancy is essential for elucidating the true transcriptional regulatory program. The general strategy is thus to cluster similar motifs and merge motifs within each cluster to create a library of non-redundant motifs [6] (Figure 1B). Next, in order to interpret the meaning of the discovered motifs, they are compared to databases of previously characterized motifs (Figure 1C). In large-scale experiments, where the motif output set is very large, the tasks of scoring, merging and identifying motifs need to be automated. To address both the clustering and the retrieval challenges, we need an accurate and sensitive method for comparing DNA motifs.

In the literature there is an ongoing discussion regarding the best representation of DNA motifs [1], [7]–[10]. Here we use a Position Frequency Matrix (PFM), which has the benefits of being relatively simple yet flexible. A PFM is a matrix of positions in the binding site versus nucleotide preferences, where each row represents one residue and each column represents the nucleotide count at each position in a set of aligned binding sites. This representation assumes that the choice of nucleotides at different positions is independent of all other positions.

**Figure 2:**
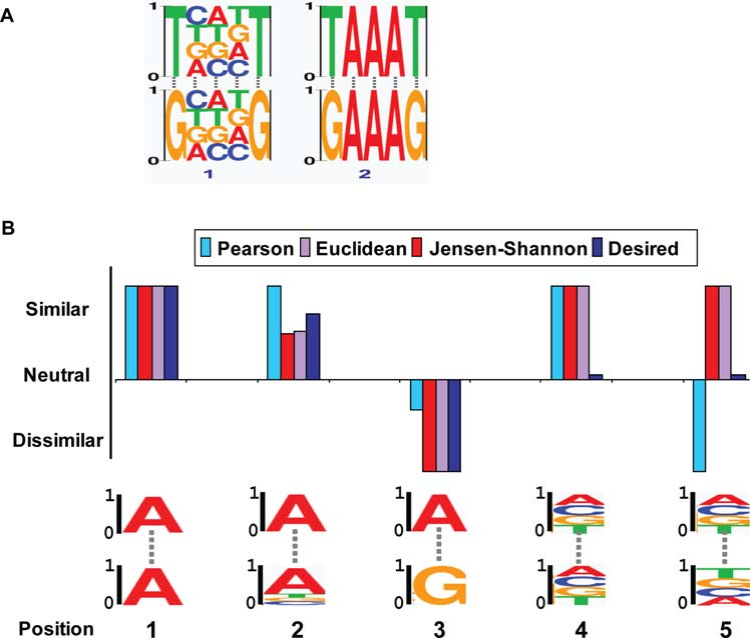
Problematic aspects of previous motif similarity scores. (A) Distinguishing between informative and non-informative positions: Two pairs of aligned motifs are demonstrated, both of which having three identical positions and two different ones. While the identical positions in the first pair (left) are non-informative, the identical positions in the second pair (right) are informative. The desired similarity score should distinguish between these two types of similarities and assign a higher score to pair number 2. The nucleotide distributions are visualized so that the height of each nucleotide is proportional to its probability (see a real life example in Figure S1). (B) Problematic aspects of motif similarity scores: The similarity score of two position frequency matrices (PFMs) decomposes into the sum of similarities of single aligned positions, due to the common position-independence assumption in the model. Here we present the similarity scores for various pairs of positions in DNA motifs according to several similarity functions, in addition to the desired score (scores are normalized to arbitrary scale of −1 to 1). The nucleotide distribution in each position is visualized as in (A) (the height of each nucleotide is proportional to its probability). As shown here, all scores (Pearson correlation, Jensen-Shannon divergence, and Euclidean distance) do not reflect the “true” similarity between two distributions or cannot differ between informative and uniform background positions. Specifically, position 1 should get a higher score than position 2, but the Pearson correlation scores for these positions are equal. Position 3 should get the lowest possible score, yet the Pearson correlation does not capture this. Both in positions 1 and 4 identical distributions are compared, but the informative position 1 should get a higher score than position 4. However, all three methods fail to obtain this. Both positions 4 and 5 analyze nearly-uniform distributions. While in position 4 two identical distributions are compared, in position 5 there are small variations, which alter the order of nucleotides. As we show, Pearson correlation grades position 5 substantially lower than position 4.

To compare two PFMs, we need to consider all possible alignments between them. Given two aligned PFMs, we utilize the position-independence assumption to decompose the similarity score into a sum of the similarities of single aligned positions. Several similarity scores can be used to compare a pair of aligned positions. One approach uses the Pearson correlation coefficient (e.g., [11],[12]). This measure, however, might inappropriately capture similarities between probabilities (Figure 2 and Figure S1). Alternative approaches are based on similarity between two distributions. This can be a metric distance, such as the Euclidean distance [13] or an information-theoretic measure, such as the Jensen-Shannon divergence [14]. While these latter distances do not have the artifacts of the Pearson correlation, they equally weight positions with similar nucleotide distributions that are specific (e.g., a strong preference for an A) and similar positions that are non-informative (e.g., identical to the background distribution) (Figure 2 and Figure S1). It is important to differentiate between these two situations: Two positions whose similarity is due to a resemblance to the background distribution are less relevant to motif similarity, as they do not contribute to sequence-specific binding of proteins [15],[16]. In this work we use this intuition to develop a novel method for comparing and merging DNA motifs, based on Bayesian probabilistic reasoning. We define a new similarity score that combines the statistical similarity between the motifs with their dissimilarity to the background distribution. To calculate this score we estimate the probabilities of DNA nucleotides in each position of the DNA motif, by a Bayesian estimator with a Dirichlet mixture prior [17],[18] to model the multi-modal nucleotide distribution at different binding site positions.

This motif similarity score is used by us to identify similar motifs that represent binding sites of the same factor and for clustering motifs. For the latter we devised a hierarchal agglomerative clustering procedure that is based on our motif similarity score. Our results show that the new method outperforms other alternatives in accuracy and sensitivity in both the clustering and retrieval tasks.

Using our new similarity score and the clustering method based upon it, we developed a large-scale analysis pipeline of DNA motif sets. This pipeline is designed for analysis following concurrent motif search by a combination of methods (using the TAMO package [19]). The goal is to process the set of DNA motifs into a set of reliable non-redundant motifs. We use our method to identify sets of DNA motifs from a large-scale ChIP-chip assay in *S. cerevisiae* [13]. This allows us to examine how transcription factors alter their DNA binding preferences under various environmental conditions and elucidate mechanisms for condition-specific preferences.

## Results

### A Novel DNA Motif Similarity Score

Our goal is to determine whether two DNA motifs represent the same binding preferences. Since the less informative positions in a motif do not contribute to sequence-specific binding of proteins, we developed a similarity score that measures the similarity between two DNA motifs, while taking into account their dissimilarity from the background distribution.

We now develop the details of the score. We can view DNA motifs as a list of binding sites from which the nucleotide distribution at each position is estimated. This view allows us to perform statistical evaluations. We assume that each binding site was sampled independently from a common distribution over nucleotides, which satisfies the position independence properties (in correspondence with the motif PFM representation described above). Then, we can evaluate the likelihood ratio of different source distributions of the sampled binding sites. In practice, we keep only the *sufficient statistics* allowing us to evaluate the likelihood of the binding sites. These sufficient statistics are the counts of each nucleotide in each position, represented as a PFM.

Our score is composed of two components: the first measures whether the two motifs were generated from a common distribution, while the second reflects the distance of that common distribution from the background (see Methods). Our Bayesian Likelihood 2-Component (BLiC) score for comparing motifs m_1_ and m_2_ is:
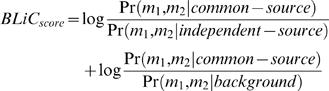
(1)Under the position independence assumption, the score decomposes into a sum of local position scores. More precisely, our likelihood-based score measures the probability of the nucleotide counts in each position of the motif given a source distribution. For two aligned positions in the compared motifs, let n^1^ and n^2^ be the corresponding positions (count vectors) in the two motifs, the similarity score is then:
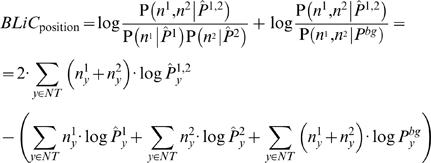
(2)where 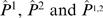 are the estimators for the source distribution of n^1^, n^2^ and the common source distribution, respectively, *P^bg^* is the background nucleotide distribution, and *NT* = {*A*,*C*,*G*,*T*}.

Since the source distribution is unknown, we must estimate it from the nucleotide counts at each position. We used a Bayesian estimator, where a priori knowledge and the number of samples were integrated into the estimation process. We considered two alternative priors. The first is a standard *Dirichlet* prior [20], which is conjugate to the multinomial distribution, enabling us to compute the estimations efficiently (see Methods). However with this prior we cannot model our prior knowledge that a position in a DNA motif tends to have specific preference to one or more nucleotides. Such prior knowledge can be described with a *Dirichlet mixture* prior [17],[18], which represents a prior that consists of several “typical” distributions. Specifically, we used a five-component mixture prior, with four components representing an informative distribution, giving high probability for a single nucleotide: A, C, G, or T. The fifth component represents the uniform distribution (see Methods).

In the above discussion we assumed that the motifs are aligned, but in practice, we compare unaligned motifs. Thus, we defined the similarity score for two motifs as the score of the best possible alignment (without gaps) between them, including the reverse complement alignment.

**Figure 3:**
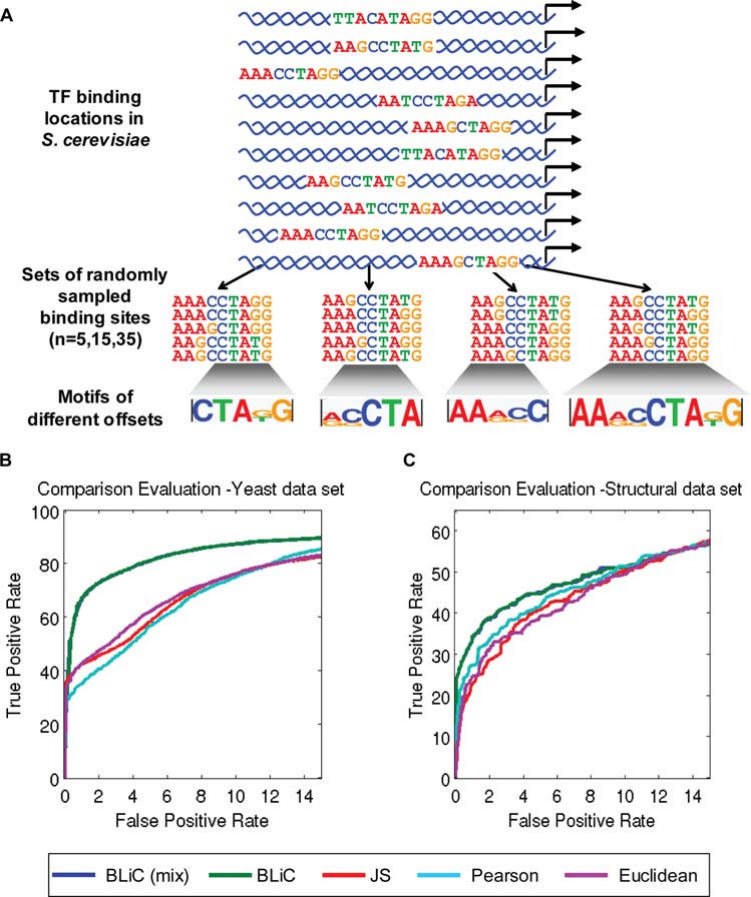
Evaluation of motif comparison scores. (A) Generating the test data set: Given a set of genomic binding sites for a transcription factor, we generate motifs by randomly sampling subsets of genomic binding sites (including 5, 15, or 35 samples per motif), aligning them, and then truncating the resulting motif to include only a part of the motif. By repeating this procedure, slightly different sets of binding sites were built for each factor. This “Yeast” data set consisted of noisy motifs for nine different *S. cerevisiae* transcription factors using the genomic sequences obtained by Harbison et al. [13], with a total of 240 motifs for each factor. (B) Sensitivity and specificity of different scoring methods: Comparison of different scoring methods on the “Yeast” data set using a subset of motifs generated from subsets of size 35 with altered lengths (not including the full length motifs, 685 motifs). Each similarity score was assigned an empirical statistical significance *p*-value. The ROC curve plots the true positive rate (TPR) vs. the false positive rate (FPR), as computed for different *p*-value thresholds, where pairs of motifs generated from genomic binding sites that were associated with the same factor are considered true positives. The BLiC score (green, using a *Dirichlet* prior, or blue, using a *Dirichlet*-mixture prior) outperformed all other similarity scores: Jensen-Shannon (JS) divergence (red), Euclidean distance (purple), and Pearson Correlation coefficient (cyan). The full arsenal of comparisons is shown in Figure S2. (C) Sensitivity and specificity estimated by structural data: Same as (B), but using the “Structural” data set of Mahony et al. [24]. Pairs of motifs from the same structural family are considered true positives.

In addition, we need statistical calibration of the similarity scores, since a high similarity score might be due to chance events [21],[22]. In particular, when comparing a single motif against motifs of different lengths, the probability of similarity by chance depends on the query motif and the length of the target. To circumvent these problems we use the *p*-value of the similarity score, which is computed empirically for each query against the distribution of scores of random motifs of a given length (see Methods and Figure 3).

#### Clustering motifs

**Figure 4:**
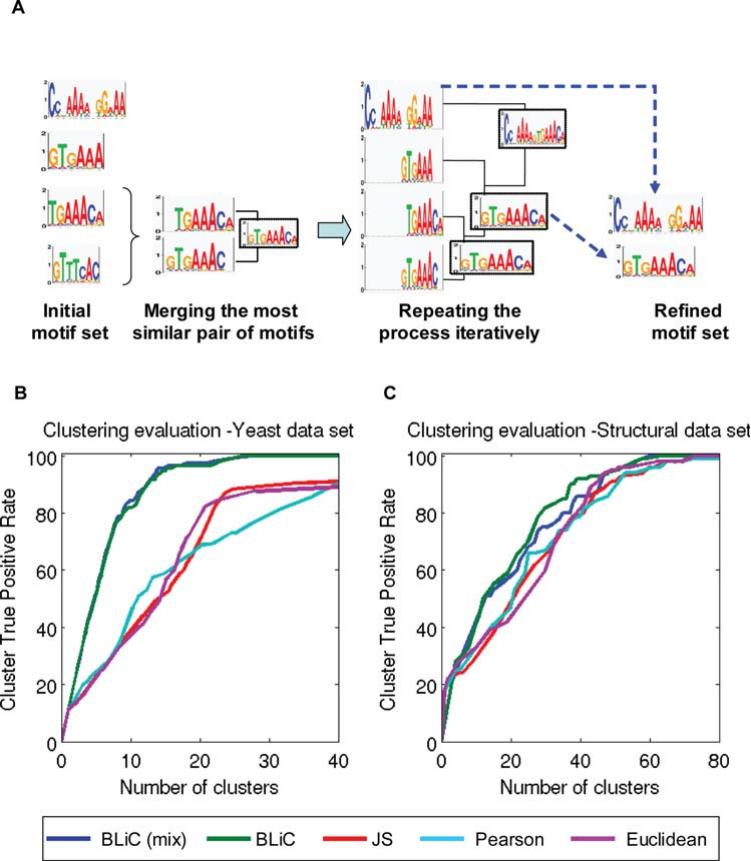
Evaluation of clustering DNA motifs. (A) Motif clustering: In this example, the initial motif set consists of four motifs. The score assigned to each pair of motifs is the score of the best possible alignment between them (including the reverse complement form, as demonstrated in this example). In each step the highest scoring pair is merged into a new motif (by combining the evidence from both motifs). These steps are repeated until we are left with a single motif. The order of merge operations results in a tree, where the leaves are the initial motifs. Each frontier in this tree creates a set of motifs. A frontier in a tree is a subset of nodes, non-descendent to each other, with every leaf in the tree a descendant of one of them. In this example, a frontier resulting in two motifs is chosen, one is an initial motif and the other is a motif created by merging three initial motifs. These two motifs are the non-redundant set of motifs, derived from the initial set. (B) Evaluation of clustering with different scoring methods: Motifs from the “Yeast” data set, generated from subsets of size 15 (180 motifs), were clustered. We split the resulting clustering tree using different thresholds. Each such threshold defines a different tradeoff between true positive rate (percent of correctly classified motifs in the clustering tree) versus the number of clusters. In this graph we plotted the average of nine repeats of clustering sets of 180 motifs described above (total of 1620 different noisy motifs). This tradeoff curve demonstrates that our BLiC score (green, using a *Dirichlet* prior, and blue, using a *Dirichlet*-mixture prior) outperforms all other scoring methods, Pearson Correlation, Euclidean distance, and Jensen-Shannon. A more detailed evaluation of clustering noisy motifs using various similarity scores is shown in Figure S3. (C) Clustering evaluated by structural data: Tradeoff curves (as in (B)) for clustering motifs in the “Structural” data set [24]. Pairs of motifs from the same structural family are considered true positives.

An important application of motif similarity scores is clustering. There are many clustering methods [23] that can be applied to motifs. Here we consider one of the simplest and straightforward clustering procedures where we combined a similarity score, such as our BLiC score, within a hierarchical agglomerative clustering algorithm. In each iteration, the algorithm computes the similarity between all pairs of motifs and then merges the most similar pair into a new motif based on the best alignment between the two motifs (see Figure 1). It then replaces the two original motifs by the new motif. These iterations are performed until we are left with a single motif. The order of merge operations results in a tree, where the leaves are the initial motifs, and inner nodes correspond to merged motifs that represent all motifs in the relevant sub-tree (see Figure 4A). We stress that this procedure is different than standard hierarchical clustering (such as UPGMA clustering). Since we merge motifs to create a new one, the similarity of the merged motif to another is different from the average similarity of each of the original motifs to that third motif.

We use the clustering tree to distill a large group of motifs into a concise non-redundant set, by splitting the tree into a subset of clusters, each representing a group of redundant motifs by choosing a frontier in the tree (see Figure 4A and Methods).

### Comprehensive Evaluation of Similarity Scores

We set out to compare our similarity score to existing ones in the literature, in the context of both motif comparison and clustering. We use two different data sets.

The first data set, which we refer to as “Yeast” is a synthetic one where we know the true labeling of motifs and use it to benchmark different procedures by relating their results with the underlying truth. To generate synthetic motifs in a realistic manner that reflects true binding properties of transcription factors, we use the genome-wide catalogue of transcription factor binding locations in *S. cerevisiae* [13]. This catalogue has high-confidence binding sites (based on combination of experimental assays with evolutionary conservation considerations). From these, we selected nine transcription factors to represent different binding patterns (in terms of inner arrangements of informative positions and length). From the binding sites of each factor we sampled sets of binding sites, and from each set generated a motif (see Figure 3A). For each factor we generated noisy motifs that differ in their quality. To do so, we varied the number of binding sites (sizes of 5, 15 or 35) and the coverage of the motif (full site, its beginning, middle, or end). We repeated this for each motif 20 times, creating a set of 240 random motifs for each of the nine transcription factors.

The second data set, which we refer to as “Structural”, was compiled by Mahony et al. [24]. Their evaluation is based on structural information. Since structurally related transcription factors often have similar DNA-binding preferences, the best match to a given motif is expected to be a motif associated with a member of the same structural class. Mahony et al. compiled a data set that contains the motifs of the families with four or more profiles in JASPAR [25].

Using these two data sets we compared different possible similarity scores for DNA motifs. Specifically, we compared the Pearson correlation coefficient; the information-theory based Jensen-Shannon divergence; the Euclidean distance; and our BLiC score.

#### Motif comparison evaluation—Identifying similar motifs

We evaluated the sensitivity and specificity of motif similarity scoring methods by comparing all possible pairs of motifs from the data sets described above, and testing whether pairs that have high similarity indeed were generated from the same source. In the “Yeast” data set we call a pair as true if the two motifs were generated from binding locations of the same transcription factor, and in the “Structural” data set we call a pair as true if the motifs are of factors from the same structural class. For each motif pair, if the similarity is statistically significant we label this as a positive pair, and otherwise call it a negative. We compared this prediction to the label of the pair, and calculated the sensitivity and specificity for each *p*-value threshold to create ROC curves (Figure 3B and 3C and Figure S2). Comparing the ROC curves of our score to those of previously suggested scores we see that the BLiC score outperformed all other scores throughout the range of possible sensitivity/specificity tradeoffs on both data sets.

The construction of the “Yeast” data set allows examining different parameters that make the task more challenging. We do so by restricting the number of binding sites or by checking whether the motif is partial or not. Using a smaller number of sites results in higher variability among motifs of the same factor, and using partial coverage means smaller overlap between compared motifs; see Figure S2. These results show that as the task becomes harder all the methods have reduced success rate: for 5% False Positive Rate (FPR), the True Positive Rates (TPR) vary from 65% (for partial overlapping motifs from samples of size 5) to 99% (for the motif with different offsets compared to the full length motifs from sample of size 35). Nonetheless, using our score improves the retrieval rates substantially in most tasks; for example, when looking at sub-motifs with partial overlap from samples of size 35, for 5% FPR using the BLiC score leads to 80% TPR, compared to 62% with the Euclidean distance or 57% with the Pearson Correlation (see Figure 3B). For some tasks, such as comparing the motifs of different offsets to the full length motifs, our method did not show statistically significant improvement (see Figure S2).

Comparing our two alternative priors, The *Dirichlet* prior versus the *Dirichlet-Mixture* prior, our results show that the more complex prior, which better models the nucleotide distribution in binding sites, leads to better results as the number of samples decreases (see Figure S2). When the number of samples is larger, the two priors result in similar performance.

#### Motif clustering evaluation—Reducing the redundancy

To further evaluate the accuracy of the different similarity scores we used these scores in clustering motifs from the two data sets. For this, we used the hierarchical agglomerative clustering algorithm described above. We then examined whether clusters consisted of motifs that are considered similar (either from the same factor in the “Yeast“ data set, or the same structural family in the “Structural” data set). Examining the cluster hierarchy at different levels of granularity we get a tradeoff curve between two criteria, the True Positive Rate (TPR) of all clusters, and the number of clusters; see Figure 4. The results show that the BLiC score outperformed the other similarity scores in the “Yeast” data set and is better than other similarity scores in the “Structural” data set.

As in the motif comparison evaluation, we can perform the clustering evaluation on various subsets of the “Yeast” data set (see Figure 4 and Figure S3). From these results we see that in harder tasks, all methods have reduced success rates. Using our score improves the clustering rates significantly when clustering all the motifs or different subsets of motifs as described above; for example, when looking at all motifs from sample sets of size 15, using our BLiC score we reach 95% TPR with less than 14 clusters, while all other do not get more than 57% TPR (see Figure 4B).

### Large-Scale DNA Motif Analyses

#### Motif analysis pipeline

**Figure 5:**
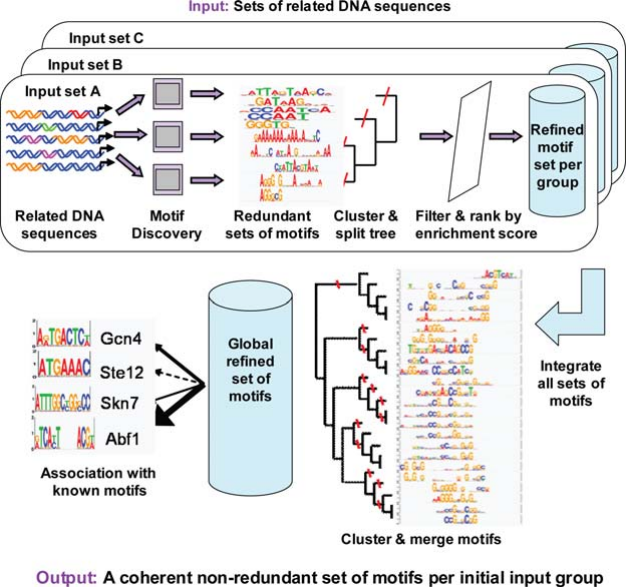
Overview of the motif analysis pipeline. The first step of the pipeline involves searching for motifs in each input set of DNA sequences, using complementary motif discovery algorithms. The motifs are filtered according to their abundance in the input set. In the second step the redundancy in the newly discovered set of motifs is reduced by clustering and merging the similar motifs. These steps are performed separately for each set (top boxes). Then, the motifs found in each input set are clustered and merged to create a global non-redundant set of motifs. These motifs are then associated with known motifs from pre-existing libraries. The refined motif set is ranked and filtered according to their abundance in each input set.

To facilitate analysis of many motifs we developed an automatic motif analysis pipeline, based on our BLiC score. This is a three-step method for processing and integrating large-scale data of newly discovered DNA motifs into coherent and reliable sets of non-redundant motifs. The inputs for this procedure are multiple groups of co-regulated DNA sequences, and the output is a set of non-redundant motifs and a ranking of their relevance for each of the input groups (Figure 5). The three steps of the pipeline include:

##### Step 1: Motif searching and filtering

We begin by applying complementary motif discovery algorithms to each group of sequences. This is done using the TAMO package [19]. Then, the newly discovered motifs undergo an initial filtration according to their abundance among the group of sequences (see Methods).

##### Step 2: Clustering and merging motifs

The integrated sets of motifs (from all input groups) are clustered and merged to create a non-redundant set. First, the discovered motifs for each group are clustered and merged separately. Then, motifs from all groups are assembled, clustered and merged. After each stage of clustering, a subset of refined motifs is automatically chosen based on the clustering tree (see Methods).

##### Step 3: Ranking and identifying motifs

Finally, the non-redundant set of motifs is ranked and filtered once again, using the abundance of the motifs in the original groups of DNA sequences (see Methods). To know which motif is new and which was previously characterized, we compare the motifs to a library of known DNA motifs from the literature (TRASFAC [26], SCPD [27], YPD [28]). By this comparison we associate the motifs with transcription factors.

### Genome-Wide Yeast Motif Library

As a real life application of this pipeline we examined genome wide ChIP-chip measurements in *S. cerevisiae* of 177 transcription factors under several environmental conditions. In total we analyzed 301 experiments for different factors and conditions [13]. We used seven motif discovery algorithms to produce a set of motifs for each ChIP-chip experiment. These motifs were clustered, filtered, ranked and compared to known motifs from the literature (as described above and in the Methods). This resulted in a concise set of DNA motifs attributed to each transcription factor under each environmental condition (all the motif sets can be found at the Supplementary Web site http://compbio.cs.huji.ac.il/BLiC).

**Figure 6:**
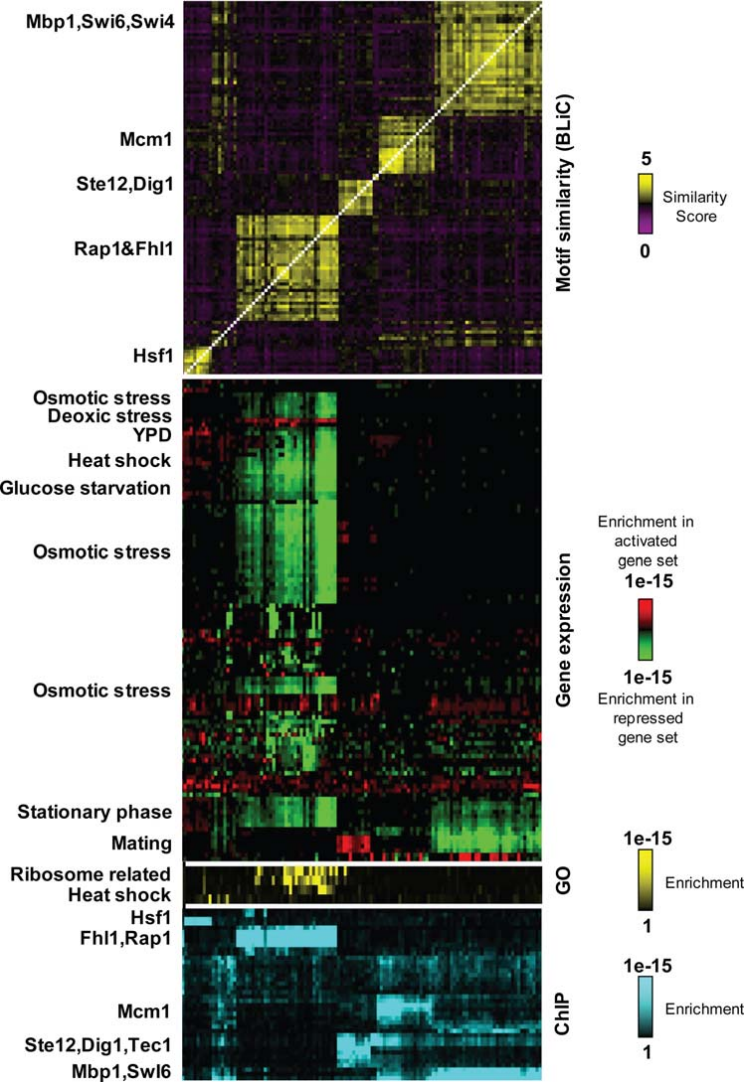
Overview of the discovered motifs. Investigation of the properties of discovered motifs. Each motif (column) is compared to other motifs using the BLiC score (rows, top square), to enrichment of putative targets among expressed or silenced genes within a compendium of gene expression at different cellular conditions (second group), to the enrichment of targets within various GO annotations (third groups) and in ChIP-chip location assays (bottom group). The rows and columns were clustered using EdgeCluster [33], an agglomerative clustering procedure that integrates various sources of information into the clustering process. Shown is clustering for partial sets of motifs related to the transcription factors: Fhl1, Sfp1, Rap1, Hsf1, Ste12, Mcm1, Swi4, Swi6, and Mbp1 (the full clustering is presented in Figure S4 and on http://compbio.cs.huji.ac.il/BLiC).

To further analyze the resulting Yeast DNA motif library, we contrast it against the wealth of genomic annotations in the yeast literature. To do so, we scanned each motif in the library against the promoters of yeast genes (see Methods) and created a target gene set for the motif. We then scored the enrichment of these motif gene sets against different types of gene annotations: the original ChIP-chip data [13], GO functional annotations [29], and groups of genes which are up or down regulated according to gene expression data (assembled by [30]–[32]). This allowed us to relate each motif to specific genomic annotations. To visualize these relationships we created a combined clustering of motifs and annotations using EdgeCluster - a clustering algorithm recently developed in our lab [33]. The novelty of EdgeCluster is in the integration of various sources of information into the clustering process. These information sources can be attributes of motifs (e.g., extent of enrichment in different gene sets) and pairwise information about motifs (i.e., the similarity of motif pairs). Figure S4 demonstrates the clustering of all the motifs. Clustering of a partial set of motifs is presented in Figure 6.

### Comparison to Previous Work

In the works of Harbison et al. [13] and MacIsaac et al. [34], the same ChIP-chip data was used to construct a global transcriptional regulatory map in yeast. The motif analyses performed in these two works differ from ours in the similarity score used (the Euclidean distance) and in the different motif clustering and merging methods. In addition, the output of these two works was a single motif for each transcription factor. To be consistent with these previous works in the comparison, we narrowed down our set of motifs for each ChIP experiment to a single motif.

**Figure 7:**
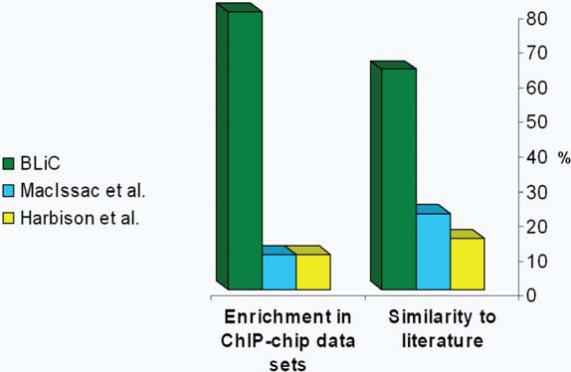
Comparison to previous analysis methods. Comparing our discovered set of motifs to the ones learned by Harbison et al. [13] and MacIsaac et al. [34]. We plot the fraction of motifs that obtained the highest score among all three sets. We first compare transcription factors with previously characterized motifs by their similarity to the known motif from the literature [26]–[28], calculated using our BLiC score. For this comparison, we took for each transcription factor the motif most similar to the known binding site (as done in these two previous works). Our motifs received the highest similarity score (among all three studies) in 65% of the cases (right). The second comparison is for transcription factors with no characterized binding motif. This comparison is based on the enrichment of the motifs in the ChIP-chip data sets. For this comparison we took the most highly enriched motif for each factor and condition (for consistency with the two previous works). The same parameters were applied in the analysis of motifs from all three methods. In this setting, our motifs were found to have higher enrichments in 80% of the cases (left).

We first looked only at transcription factors with previously characterized motifs. Our criterion for comparison is measuring the similarity to known motifs from the literature (TRANSFAC [26], SCPD [27], YPD [28]), using our BLiC score. To narrow down our motif set to a single motif for each factor we chose (as done in these previous works) the motif most similar to the known motif. In 65% of the cases our motifs have the highest similarity to the known motifs (Figure 7, Table S1). The motifs learned by the algorithms of MacIsaac et al. and Harbison et al., had the highest similarity only in 22% and 12% of the motifs, respectively.

For transcription factors with no previously known binding motif in the literature, we compared the enrichment of the motifs within the ChIP-chip groups of sequences. For the comparison, we narrowed the motif sets by choosing the most significant motif for each factor and environmental condition (similarly to what was done in these previous studies). We scanned the genomic sequences and computed the enrichment of each motif (see Methods), using the same procedure and parameters for motifs from all three methods. Our motifs were found to have the highest enrichments in 80% of the cases (see Figure 7 and Table S1).

To ensure that the improvement we see is not due to differences in motif discovery methods, we repeated the analysis using the original output of the motif discovery of Harbison et al. (data not shown). This lead to slight changes in the output motifs, as our original analysis used a superset of these motifs. Comparing these modified results against the results of Harbison et al. and MacIssac et al. we see essentially improvement as the one we reported above (in 62% of the cases our motifs have the highest similarity to the known motifs, and in 65% of the cases our motifs were found to have the highest enrichments).

### Elucidating Conditional Binding of Transcription Factors

Using the motif sets we have learned, we next turned to examine the change in the binding specificities of the transcription factors under different conditions. We distinguish between two types of factors. A *condition-independent* factor binds the same targets in multiple conditions, while a *condition-dependent* factor changes its set of targets between conditions. An example of a condition-independent transcription factor in yeast is Fhl1, a master regulator of ribosomal genes, which according to the ChIP data remains bound to 75% of its targets under different conditions (see Figure S5A). This is consistent with previous work [35] and with the motif analysis, where similar motifs are related to Fhl1 in all three conditions (see Figure S5B).

A condition-dependent regulator can show a range of behaviors in response to a change in condition. It may expand and bind additional targets, it may alter and bind to a different set of targets, or it may even not bind any targets [13]. Various mechanisms may be involved in monitoring condition-dependent binding. A factor may expand its targets, due to dosage change of the active transcription factor in the nucleus [13]. Alternatively, a factor may alter its targets due to several probable mechanisms (see Figure S6). One mechanism is changing the factor's specificity to the DNA, which we can trace by identifying variations in the DNA motif (Figure S6A). Another possible mechanism is a change in the factor's binding partner, which may be detected through co-occurrence of motifs of different factors (Figure S6B). In addition, a change of targets may be caused by a change in the accessibility to the binding site, which we cannot identify by analyzing motifs (Figure S6C).

We focus here on factors that alter their targets under different conditions and try to elucidate the mechanism. We defined a transcription factor as altering its target genes between two conditions, if the number of target genes in the intersection is less than half of the number in each condition separately. In addition, we considered only factors with at least 20 target genes in each of the two conditions (a sufficient number for motif discovery). Out of the 72 transcription factors for which ChIP-chip experiments were carried out in more than one condition, 50 factors alter their target genes between two conditions (in total, 112 pairs of differential conditions) (Table S2). We searched for differential motifs in the motif set of each factor at every condition. We say a motif is differential if there is a significant difference (p<0.05, chi-square test) in the fraction of ChIP targets containing the motif between the two conditions (excluding the genes in the intersection). This analysis can potentially elucidate the mechanism through which a factor changes its DNA targets, by finding different variants of motifs, or co-occurrence of motifs of different factors as explained above. In about half of these pairs we did not find statistically significant motifs in at least one of the compared conditions and thus could not search for differential motifs. Finding a motif only for one condition could be meaningful on its own, since this may indicate that in the other condition there is no direct binding of the factor to the DNA. On the other hand it could result from technical reasons, such as noise in the input set of sequences, and thus in this work we do not analyze these cases. Out of the remaining 52 pairs (spanned over 27 different transcription factors), we found differential motifs for 88% of the factors (47 cases spanned over 24 factors, see Table S3) with a *p*-value of less than 0.05.

### Condition-Dependent Binding of Ste12 under Conditions of Mating and Filamentous Growth

**Figure 8:**
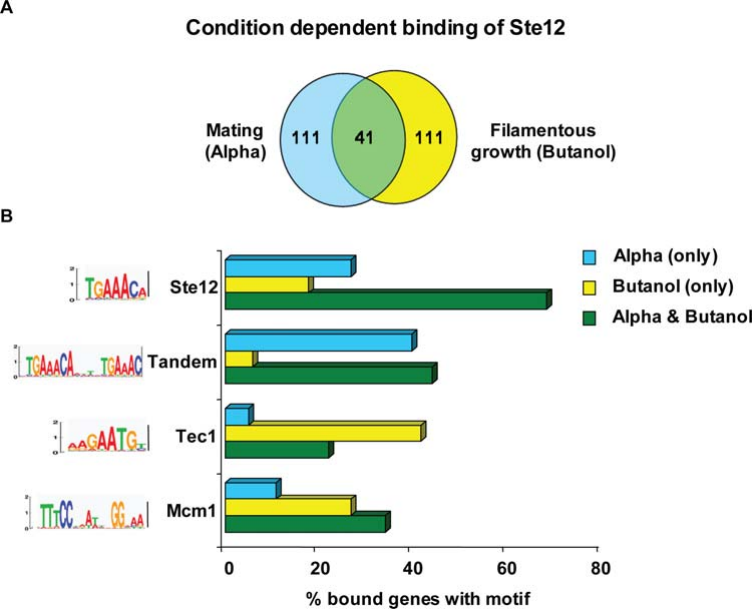
Condition dependent behavior of Ste12. (A) A Venn diagram representing the results of the ChIP-chip experiment [13] for Ste12 under mating (induced by alpha factor) and filamentous growth (induced by butanol). Ste12 alters its targets substantially between these two conditions. (B) Analysis of the percent of sequences bound by Ste12 which contain the different motifs (when searching for motif occurrences at 2% false positive rate). Shown are the different motifs in the targets bound by Ste12 in filamentous growth condition only (yellow), in mating condition only (blue), or in both conditions (green). Each motif is shown as a sequence logo on the left and percent occurrence in each group as bar chart on the right. We can see that under filamentous growth there is enrichment for a motif similar to the previously characterized Ste12 motif (top motif), as well as the known recognition sequence of Tec1 (third from top). Under mating there is an enrichment for a near-perfect tandem repeat of Ste12 known binding site (second from top). A motif similar to the known Mcm1 motif (bottom motif) is found to be enriched under both conditions, especially under filamentous growth.

An example of a transcription factor that shows condition-dependent binding is Ste12, which activates genes in two alternative pathways—mating and filamentous growth [36],[37] (Figure 8A). Under filamentous growth signaling (Butanol induction) we found that Ste12 binds promoters enriched with its known motif [38], as well as the known recognition sequence of Tec1 [38], a co-factor that binds the DNA with Ste12 under filamentous growth [37],[39] (Figure 8B). However, under mating conditions (Alpha factor induction) we find that Ste12 binds promoters with another variant of the motif more highly enriched than the known one. This variant is a near-perfect tandem repeat of its known site, suggesting that Ste12 binds the DNA as a homodimer following Alpha factor induction [40],[41] (Figure 8B). An additional player found in our analysis is Mcm1, whose known motif [42] is enriched among promoters bound by Ste12 under both conditions. This is consistent with the role of Mcm1 inhibiting expression of mating genes in diploid cells [42]. Mcm1 may play a similar role in the filamentous growth pathway, in which haploid cells undergo invasive growth, and diploid cells undergo pseudohyphal growth. Interestingly, the exact same motifs were learned for the ChIP targets of the cofactor Dig1, under all the conditions stated above, which indicates that Dig1 does not bind the DNA directly [37]. Thus, looking at the discovered motif sets, we can reveal the regulators involved and propose a mechanism through which a transcription factor alters its targets under different conditions. Here we propose the altered binding is caused by a change in the DNA binding partner: Ste12 binds the DNA with Tec1 under filamentous growth and as a homodimer under mating conditions.

### Condition-Dependent Binding of the Iron-Regulated Factor Aft2

**Figure 9:**
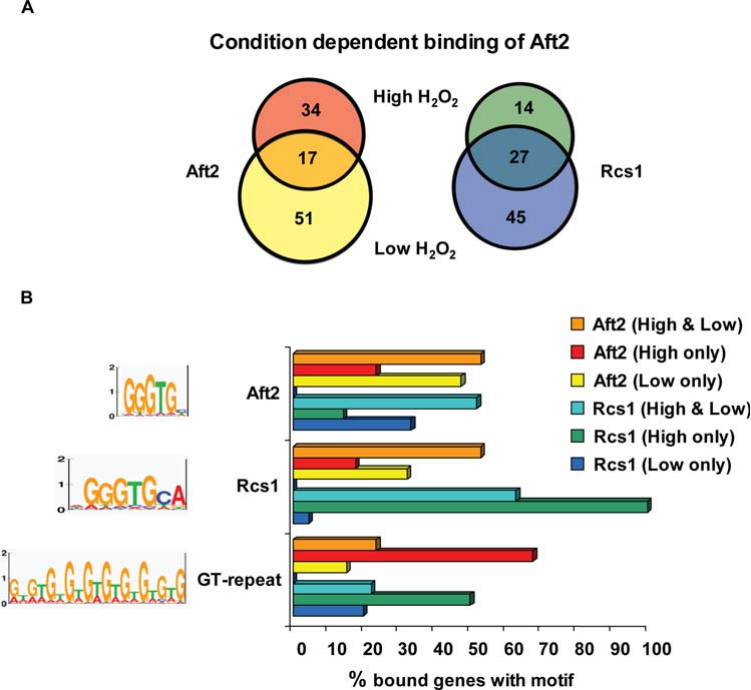
Condition dependent behavior of Aft2. (A) Venn diagrams representing the results of the ChIP-chip experiment [13] for the transcription factors Aft2 and Rcs1 under high and low H_2_O_2_ stress. Aft2 alters its targets substantially between these two conditions. (B) Analysis of percent of sequences bound by Aft2, which contain the different motifs (when searching for motif occurrences at 2% false positive rate). Shown are the different motifs in the targets bound by Aft2 and Rcs1 in low H_2_O_2_ stress only (yellow and blue, respectively), in high H_2_O_2_ stress only (red and green, respectively) or in both conditions (orange and cyan, respectively). Each motif is shown as a sequence logo on the left and percent occurrence in each group as bar chart on the right. Under low H_2_O_2_ stress there is enrichment for a motif similar to the previously characterized Aft2 motif (top motif), as well as for the known recognition sequence of Rcs1 (middle motif). Under high H_2_O_2_ stress only abundant low-complexity repeats of Poly-GT (bottom motif) have been identified.

Another interesting example is provided by the iron-regulated transcription factor Aft2, required for iron homeostasis and resistance to oxidative stress [43]. This factor exhibits a significant environmental-dependent binding, switching targets between low and high H_2_O_2_ conditions (Figure 9A). The role of Aft2 in iron homeostasis and resistance to oxidative stress is poorly understood. In low H_2_O_2_, we find that Aft2-bound promoters are highly enriched with a motif similar to the known recognition sequence of Aft2 (GgGTG) [43]. However, in high H_2_O_2_ we find abundant occurrences of a low complexity Poly-GT motif (Figure 9B). This result indicates that a possible explanation for the change in Aft2 DNA targets is a change in its DNA binding specificity over these conditions. We reach this conclusion due to the lack of the known motif or motifs of other factors in the bound targets under high H_2_O_2_ and due to the similarity of the Poly-GT to the known motif. Furthermore, the poly-GT motif under high H_2_O_2_ may suggest that Aft2 binds the DNA as a homodimer. Interestingly, the known motif of Aft1 (Rcs1) [43], a paralog of Aft2, was enriched among the Aft2-bound promoters in low H_2_O_2_ condition. This implies a possible overlap between the targets of Aft2 and Aft1, supported by ChIP-chip data of the two factors (Figure 9B). Based on our analysis, we report two similar (but not identical) motifs for the two paralogs (as suggested by [43],[44]). Since it is known that Aft2 and Aft1 have independent and partially redundant roles in iron regulation [43],[44], this strengths our assumption that Aft2 binding to the DNA does not depend on Aft1, but is due to a change in its specificity to the DNA. The ChIP-chip data and our motif analysis suggest that under high H_2_O_2_ conditions Aft2 has a unique role in gene regulation. Here again, by looking at the motif sets, we propose a mechanism for condition dependent binding of a transcription factor. In this case we propose the cause is a change in the factor's specificity to the DNA.

## Discussion

An accurate motif comparison method is important for clustering redundant DNA motifs into coherent groups and for connecting the discovered motifs to previously characterized motifs. In this study we present a novel similarity score, the BLiC score, based on Bayesian probabilistic principles. We use the new comparison method as a basis for motif clustering and retrieval procedures, and compare it to several commonly used alternatives. This comparison shows that our BLiC score improves the specificity and sensitivity of motif comparisons and clustering tasks. The resulting motif clustering and retrieval procedures are incorporated in a large-scale automated pipeline for analyzing DNA motifs, which integrates the output of various DNA motif discovery algorithms and automatically merges redundant motifs from multiple training sets. The output of our pipeline is a coherent annotated library of motifs. Application of this pipeline to genome-wide location data of transcription factors in *S. cerevisiae*, successfully identified DNA motifs in a manner that is as good as semi-automated analyses reported in the literature. Moreover, we demonstrate how motif analysis can lead to insights into regulatory mechanisms.

### Hierarchical Agglomerative Clustering

We used our BLiC score to develop a hierarchical agglomerative clustering algorithm for merging similar motifs, in which we ensure that the motifs within every sub-tree are properly aligned. Furthermore, such an approach allows us to trim the cluster tree at various levels, thus allowing us to merge motifs at different resolutions. In our method a new agglomerative node results from aligning and merging the motifs of its descendent nodes, and then computing the similarly of this new motif to all other nodes. As a consequence, the hierarchical progression ensures that each sub-tree is coherent. This is in contrast to many clustering methods, such as k-means and typical hierarchical clustering [45] which find a set of motifs that are all similar to each other, but are not necessarily coherent in the sense that they cannot all be aligned.

### Motif Analysis

Our motif analysis pipeline is designed to process discovered DNA motifs into a set of non-redundant motifs and compare these with known motifs. As we have shown, our approach improves the sensitivity and specificity in the analysis of the outputs of standard motif discovery methods. By automating all the steps, we enable the analysis of hundreds of input groups. In addition, we achieve a wide view on transcription regulation by running several motif discovery algorithms in parallel, and integrating their outputs. By comparing motifs from different input groups we are able to connect between transcription factors that play a role in different processes. Our analysis does not focus on finding the “best” single motif for each input group (e.g., targets of ChIP-chip assay), but rather we find a set of non-redundant motifs and their relations (enrichment) to each input group. This output better captures the complexity of the underlying regulatory program. For example, in many cases we find motifs of co-factors (e.g., Ste12 and Tec1). In other cases we see that a factor changes its binding specificity under different conditions (e.g., Aft2). For these cases, several DNA motifs better capture the DNA binding preferences of the transcription factor than a single motif.

### Relations to Previous Work

There are several different approaches attempting to quantify similarities between DNA motifs. Two previous works [21],[22] showed that using *p*-values when comparing motifs is more accurate than the actual similarity scores. Specifically, Gupta et al.[21], compared seven motif-motif position similarity functions, including the Pearson Correlation coefficient (e.g., [11],[46]), average log-likelihood ratio (ALLR) [16], Kullback-Leibler divergence [47]–[49], and the Euclidean distance (ED) [13],[50]. They found that the Euclidean distance is slightly better than the alternatives they considered. The data set used by Gupta et al. has a similar design as our data set, but it is based on the TRANSFAC database [26]. Not surprisingly, our results are consistent with theirs. Here we also use *p*-values to calibrate similarity scores, and show that our score is more accurate than the Euclidean distance, which is the second best.

Several resources are available for DNA motif analysis. There are many open access motif discovery tools available (e.g., [2],[3],[11]) and motif comparison tools [11],[21],[51]. In addition there are several available tools that integrate multiple motif discovery tools, and supply additional tools for filtering, comparison and ranking motifs [19],[49],[52]. In our motif analysis pipeline we use the TAMO package [19], for motif discovery and filtering, with a different genomic scan approach using statistical tools [53]. The main difference is that for the motif comparison and clustering we use our new BLiC score and a hierarchical agglomerative clustering (as discussed above).

### From DNA Motifs to Regulatory Mechanisms

Sequence information is a highly accessible resource, and thus it is interesting to ask whether it can help elucidate mechanisms of transcription regulation. We examined transcription factors that alter their targets in response to an environmental change, and found a differential motif in 88% of these cases (24/27 factors). These differential motifs can suggest the potential mechanism through which the factor changes its targets. We show that motifs provide an indication for potential mechanisms when the factor changes its binding partner (Figure S6A) or its specificity to the DNA (Figure S6B), as we discussed thoroughly for the case of Ste12 and Aft2. Nevertheless, motif analysis obviously does not reveal the whole regulatory picture. For example, chromatin-modeling mediated regulation cannot be inferred from motif analysis (Figure S6C). Thus, for a complete understanding of the regulatory mechanisms additional information is needed.

A significant limitation of motif analysis in general, is the discrepancy between putative binding sites and actual functional binding events. This raises the question addressed frequently before [10],[54], whether our representation of transcription factor binding preferences is sufficiently accurate.

In this study we overcome a basic obstacle in DNA motif analysis, by developing an accurate motif comparison method. Our motif analysis pipeline, which includes clustering and retrieval procedures based on our novel score, is fully automated and produces accurate results. This is highly important in large-scale analysis, such as the one reported here. We showed the power of motif analyses, which is useful not only for building regulatory maps, but also for understanding more profoundly regulatory mechanisms.

## Methods

### Motif Representation

We use a Position Frequency Matrix (PFM) representation for a DNA motif. This is a n×4 matrix, where each i,j cell contains the count of nucleotide j in position i of the motif.

### Scores

We define the similarity score for two aligned PFMs. Due to the positional independence assumption in PFMs, the score decomposes into the sum of scores for corresponding positions. Our score is composed of two components: The first measures whether the two motifs were generated from a common distribution. The second reflects the distance of that common distribution from the background. Thus, for positions n^1^ and n^2^, our score is as described in Equation 2. Statistically, in the score we sum the log-likelihood-ratio of two pairs of hypotheses.

The first component:

**H_0_**: The two samples were drawn from a common source distribution.

**H_1_**: The two samples were drawn independently from different source distributions.

The second component:

**H_0_**: The two samples were drawn from a common source distribution that is distinct from the background.

**H_1_**: The two samples were drawn from the background distribution.

### Estimation

We estimate the source distributions from the PFM using a Bayesian approach, with a *Dirichlet* prior. The *Dirichlet* prior is specified by a set of *hyper-parameters* α = (α_1_,α_2_,…α*_n_*) and has the form: 
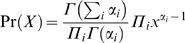


Where Γ(*x*) is the *Gamma function.* We use two prior variants: The first is a standard *Dirichlet* prior [20], with hyper-parameters of (1,1,1,1). When using this prior, the estimated distribution for position n is:
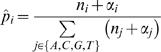
where α is the vector of hyper-parameters.

The second prior we use is a five-component *mixture of Dirichlet* prior [17]. We merge five Dirichlet priors using uniform weights. Four of the components give high probability for a single DNA nucleotide: A, C, G, or T. The fifth element represents the uniform distribution. We use the hyper-parameters (5,1,1,1) for A, (1,5,1,1) for C, etc., For the fifth component we use the hyper-parameters (2,2,2,2). Using this, the estimated distribution for position n is:
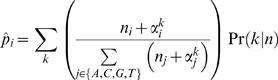
This is a weighted average, where the weights are the posterior probabilities of each component given the data. The posterior is: 
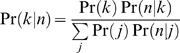
where, 
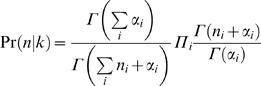



### Clustering and Comparing Motifs

#### Comparing motifs

The similarity score of two PFMs is the score of the best possible alignment (without gaps) between them, including the reverse complement alignment. The unaligned flanks of the motif are scored according to their distance from the background distribution multiplied by a relaxing factor of 0.2.

#### Assigning *p*-values to motif similarity scores

We devised an empirical *p*-value estimation procedure for motif similarity scores. For each motif, we computed the score distribution against motifs of all possible lengths, by comparison to 1000 random motifs of a specified length. Since the BLiC score distribution depends on the specificity of each motif, the distribution is computed for each motif separately to retain the overall characteristics of the motif. The random motifs were generated by sampling positions of motifs from the TRANSFAC database [26]. The *p*-value of the similarity of a given DNA motif to another, is calculated empirically from the score distribution of the first motif against random motifs of the same length as the second motif (calculating the fraction of random motifs that got the same score or higher).

### Clustering and Trimming the Tree

To cluster motifs, we implemented a hierarchical agglomerative clustering algorithm, using various motif comparison scores. In each iteration, the algorithm computes the similarity between all pairs of motifs and then merges the pair with the highest similarity score into a new motif (see Figure 4A). This merge includes aligning the motifs according to the best scoring alignment between them, and then combining the evidence from both of them, by summing their nucleotide counts at each position (i.e., the motifs are weighted according to their number of samples). These iterations are repeated until we are left with a single motif. The order of merge operations results in a tree, where the leaves represent initial motifs, and each inner node represents the merging of all original motif sub-tree below it.

The clustering tree is used to distill the input set into a non-redundant group, by splitting the tree into clusters representing groups of redundant motifs. To obtain this non-redundant set, which covers the initial set, we choose a frontier in the clustering tree. A frontier in a tree is a subset of nodes, non-descendent to each other, where every leaf in the tree is a descendant of one of them. This is done by a bottom-up traversal over the tree in which we choose the set of nodes in the required frontier. Specifically, we consider every two motifs that were merged into one in the tree. We want to identify situations where this merge resulted in a motif that is very different than each of the two motifs that were merged. To test that, we compare the degree of similarity between the two motifs to the maximal score we could have attained (the maximum of the similarity of each one to itself). If the observed score's ratio to this maximum is less than a preset threshold, the two motifs are added to the frontier. In the motif analysis pipeline, we use a stringent threshold of 60% of the maximum for creating non-redundant motifs (chosen according to hand-curated splits of 10 trees).

### Motif Analysis

#### Motif discovery algorithms

In the analysis pipeline we applied several motif discovery algorithms—MDScan [2], AligneAce [11], and MEME [3] were used through the TAMO package [19], with the default parameters (apart from the MEME algorithm, for which we changed the parameters to output six motifs). We included conserved and abundant motifs in the yeast genome [55], and the output of MEME_c [13], Converge [13] and the SeedSearcher motif discovery algorithm [56]. The discovered motifs underwent an initial filtration according to their enrichment among the initial group of sequences (*p*-value threshold of 10^−5^, calculated using the TAMO package [19]). All motifs are converted to a PFM representation.

#### Clustering motifs

In the second step of the pipeline we cluster the motifs—first we clustered the motifs discovered for each transcription factor under each environmental condition separately, then the (merged) motifs for each factor under all conditions, and finally the entire set of motifs. The motifs are clustered and merged as described above.

#### Truncating motifs

Uninformative positions at the two edges of motifs were truncated automatically. This was done by a chi-square test (threshold of 0.05), testing if the nucleotides at a motif position distribute according to the background.

#### Identifying the motifs

Connecting between discovered motifs and transcription factors, we compared the motifs against a set of known motifs (TRANSFAC [26], SCPD [27], YPD [28]).

#### Ranking motifs

In the third step of the pipeline, we rank and filter the merged motifs according to their enrichment (−log hyper-geometric *p*-value) in the input groups of DNA sequences. For filtering we use a threshold of 3 after applying a Bonferroni correction for multiple hypotheses. For this, we find the occurrences of each motif using a statistical tool for genomic scan, TestMotif program [53]. To scan the genome with our motifs, we transferred them from PFMs (count matrices) to profiles (frequencies), using estimation with *Dirichlet-mixture* prior described above. After scanning with the TestMotif program [53], we combine evolutionary conservation data to find the occurrences of motifs. Particularly, we decide whether a DNA sequence contains a motif if one of two following criteria holds:

- The sequence contains a highly statistically significant binding site, using a *p*-value threshold of 0.03 after Bonferroni correction for multiple hypotheses according to the average length of the scanned sequences (a good sequence match between the motif and the binding site).
- A less statistically significant occurrence of the motif (threshold of 0.1), highly conserved among seven species of the genus *Saccharomyces* (average conservation of the motif is at least 0.6, according to the UCSC conservation track (phastCons [57], through the UCSC Genome Browser Database [58]).

#### Parameter tuning

The threshold values listed above were chosen according to an extensive search of parameters that maximize the true positive rate, allowing up to 2% false positive calls. This optimization was based on location analysis data of Gcn4 [13], and location and expression data for Sko1 (unpublished data).

## Supporting Information

Figure S1 Distinguishing between informative and non-informative positions: Two pairs of aligned motifs are presented (by a sequence-logo). This is an alignment of the known motif for the invertebrate factor Dfd versus two variants of the vertebrate factor Pax4, all taken from TRANSFAC [26] (matrix accessions I$DFD_01, V$PAX4_02, and V$PAX4_04, all from version 8.3). While it is clear the first motif (left) should get a lower similarity score than the second motif (right), scoring the two pairs of aligned motifs using the Jensen-Shannon divergence yields a higher score for the first motif. The desired similarity score should distinguish between high similarity of informative positions and non-informative positions.
(0.73 MB TIF)

Figure S2 Evaluation of motif comparison. Using different subsets of motifs out of the “Yeast” data set, we compare our BLiC score (green, using a Dirichlet prior, and blue, using a Dirichlet-mixture prior) with other similarity scores: Jensen-Shannon divergence (red), Euclidean distance (purple) and Pearson Correlation coefficient (cyan). Each of the nine panels represents a different comparison. The columns correspond to the number of samples used for constructing the motifs. The rows correspond to different choices of query sets and target sets for comparison (illustrated by the logos on the right): In the top row all motifs of partial offsets are queries against the same set. In the middle row, all motifs, including full-length motifs and partial offsets are compared against themselves. In the bottom row, we use partial offset motifs as queries and full motifs as targets. In each panel we plot True Positive Rate (y-axis) vs. False Positive Rate (x-axis) as in Figure 3B.
(1.67 MB TIF)

Figure S3 Evaluation of motif clustering. Using different subsets of motifs out of the “Yeast” data set, we compare our BLiC score (green, using a Dirichlet prior, and blue, using a Dirichlet-mixture prior) with other similarity scores: Jensen-Shannon divergence (red), Euclidean distance (purple) and Pearson Correlation coefficient (cyan). Each of the nine panels represents the average performance of 9 repeats of clustering over different motif sets. The columns correspond to the number of samples used for constructing the motifs. The rows correspond to different choices of motif sets (illustrated by the logos on the right): In the top row we cluster all motifs of partial offsets. In the middle row, we cluster all motifs, including full-length motifs and partial offsets. In the bottom row, we cluster only full motifs. In each panel we plot True Positive Rate (y-axis) vs. number of clusters (x-axis) as in Figure 4B.
(1.88 MB TIF)

Figure S4 Overview of the discovered motifs. Investigation of the properties of discovered motifs. Each motif (column) is compared to other motifs using the BLiC score (rows, top group), to average expression of its targets in different experiments [30]–[32] (second group), to enrichment of its targets in GO annotations [29] (third groups) and in ChIP-chip location assays [13] (bottom groups). The rows and columns were clustered using the EdgeCluster [33] algorithm, which integrates various sources of information into the clustering process. These information sources are attributes of motifs and pairwise information about motifs. The results are clusters of motifs that have not only similar attributes, as in regular clustering algorithm, but also similar relations to motifs in other clusters.
(8.43 MB TIF)

Figure S5 Condition independent binding of Fhl1. (A) A Venn diagram representing the results of the ChIP-chip experiment [13] for the transcription factor Fhl1 under YPD conditions, amino-acid starvation and nutrient deprived conditions. The targets of Fhl1 do not change under these three environments. (B) Under all conditions the same motif is found to be highly enriched.
(2.70 MB TIF)

Figure S6 Possible mechanisms for condition-dependent binding of TFs. Motif analysis for condition-dependent transcription factors that bind different targets under different conditions. Here, three possible mechanisms that may be involved in monitoring condition-dependent binding, which lead to altered targets, are presented schematically. For each mechanism we show the scheme of the promoter organization of the target genes (above the dashed line) and the result of motif discovery (under the dashed line). (A) The first mechanism is through a change in the cofactor. This may be detected through co-occurrence of motifs of different factors. (B) The second mechanism is through a change in the specificity to the DNA. This change can be traced by identifying variations in the DNA motif. (C) The third mechanism is a change in the chromatin state. This change cannot be traced using motif analysis.
(0.98 MB TIF)

Table S1 Comparison of the Yeast motif library to previous works
(0.02 MB XLS)

Table S2 Transcription factors with condition-dependent binding which alter their targets.
(0.02 MB XLS)

Table S3 List of differential motifs.
(0.04 MB XLS)
