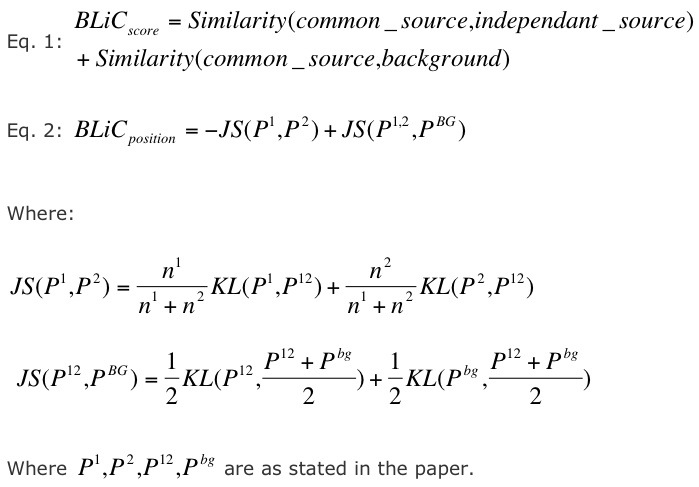# Correction: A Novel Bayesian DNA Motif Comparison Method for Clustering and Retrieval

**DOI:** 10.1371/annotation/d876137b-59c5-48cf-8491-c8cf12f26a9b

**Published:** 2011-05-09

**Authors:** Naomi Habib, Tommy Kaplan, Hanah Margalit, Nir Friedman

In the Results section the two main mathematical expressions, Equations 1 and 2 in the section titled "A Novel DNA Motif Similarity Score" are incorrect. The authors explain that their BLiC score is described using a log-likelihood ratio test. An equivalent way of writing this score is using the Kullback-Leibler (KL) divergence, thus measuring the distance between the described estimated probabilities (the common source distribution, independent source distribution, and the background distribution). In their implementation of the score, the authors used the Jensen-Shannon distribution, a symmetrical version of the KL divergence measure. A corrected version of this score can be viewed here: