# Supplementary figures and images for: A Novel Bayesian DNA Motif Comparison Method for Clustering and Retrieval
Source: PLoS Comput Biol. 2008 Feb 29;4(2):e1000010. doi: 10.1371/journal.pcbi.1000010 (PMC2265534; doi:10.1371/journal.pcbi.1000010)

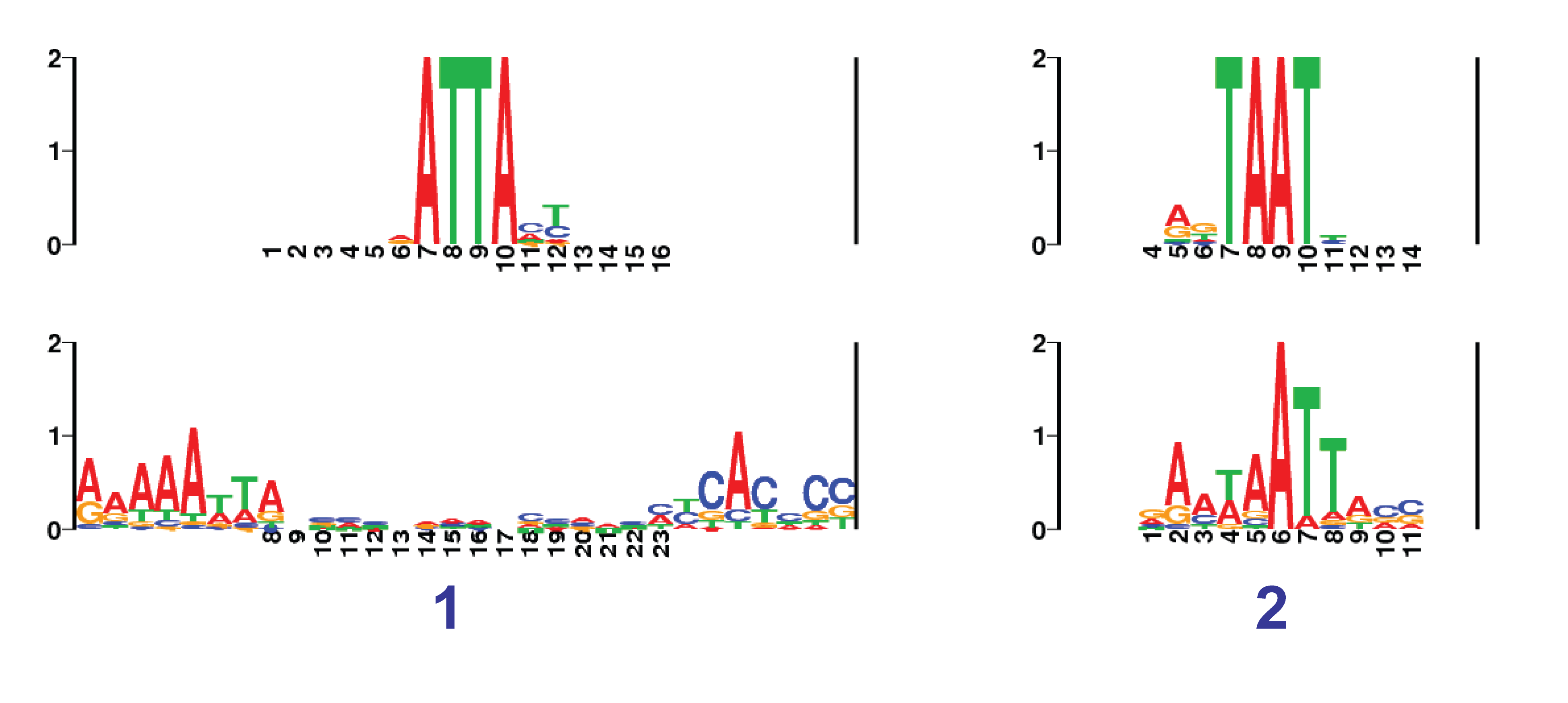

Supplement: Figure S1 — Distinguishing between informative and non-informative positions: Two pairs of aligned motifs are presented (by a sequence-logo). This is an alignment of the known motif for the invertebrate factor Dfd versus two variants of the vertebrate factor Pax4, all taken from TRANSFAC [26] (matrix accessions I$DFD_01, V$PAX4_02, and V$PAX4_04, all from version 8.3). While it is clear the first motif (left) should get a lower similarity score than the second motif (right), scoring the two pairs of aligned motifs using the Jensen-Shannon divergence yields a higher score for the first motif. The desired similarity score should distinguish between high similarity of informative positions and non-informative positions. (0.73 MB TIF) [file pcbi.1000010.s001.tif]

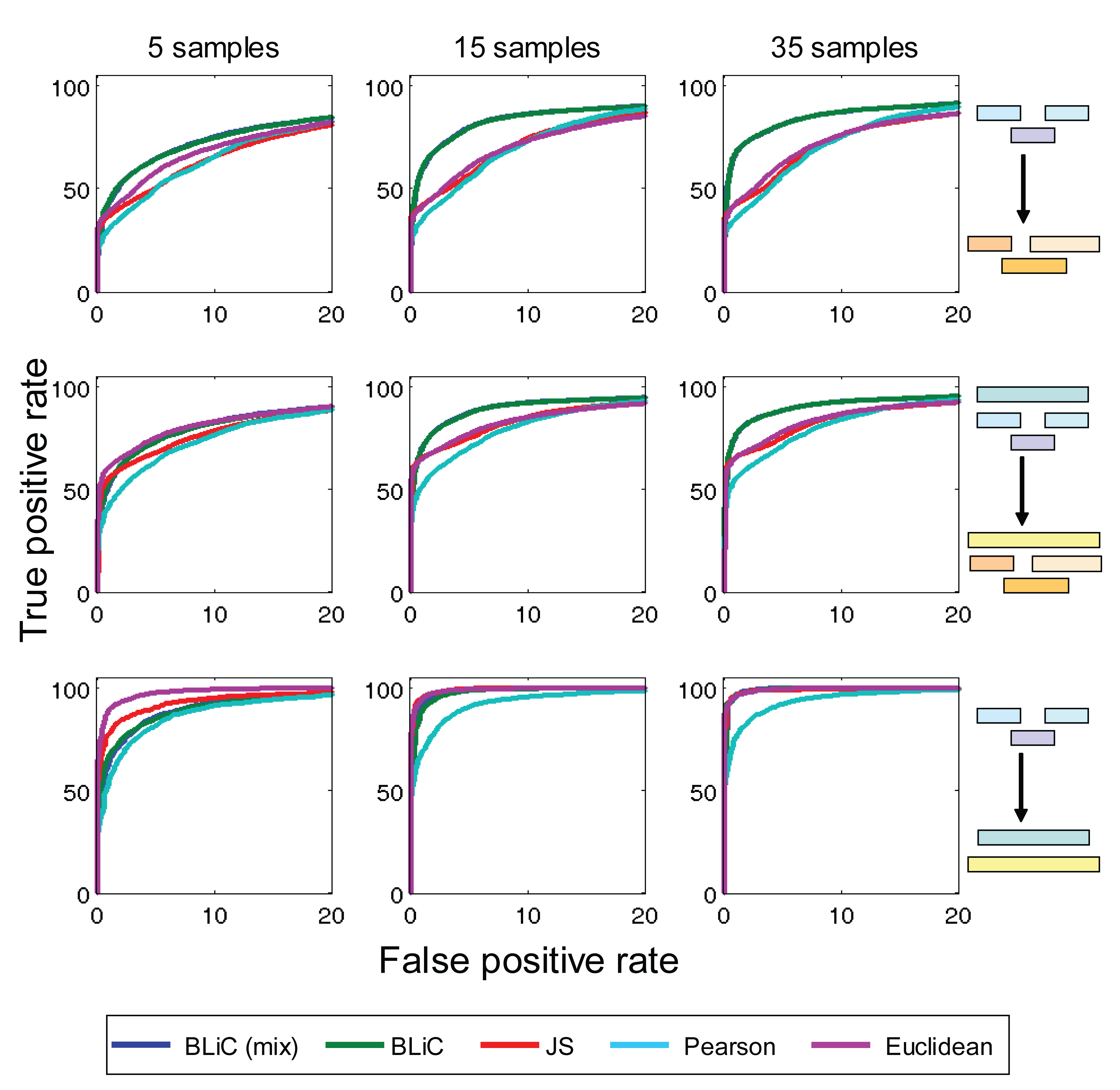

Supplement: Figure S2 — Evaluation of motif comparison. Using different subsets of motifs out of the “Yeast” data set, we compare our BLiC score (green, using a Dirichlet prior, and blue, using a Dirichlet-mixture prior) with other similarity scores: Jensen-Shannon divergence (red), Euclidean distance (purple) and Pearson Correlation coefficient (cyan). Each of the nine panels represents a different comparison. The columns correspond to the number of samples used for constructing the motifs. The rows correspond to different choices of query sets and target sets for comparison (illustrated by the logos on the right): In the top row all motifs of partial offsets are queries against the same set. In the middle row, all motifs, including full-length motifs and partial offsets are compared against themselves. In the bottom row, we use partial offset motifs as queries and full motifs as targets. In each panel we plot True Positive Rate (y-axis) vs. False Positive Rate (x-axis) as in Figure 3B. (1.67 MB TIF) [file pcbi.1000010.s002.tif]

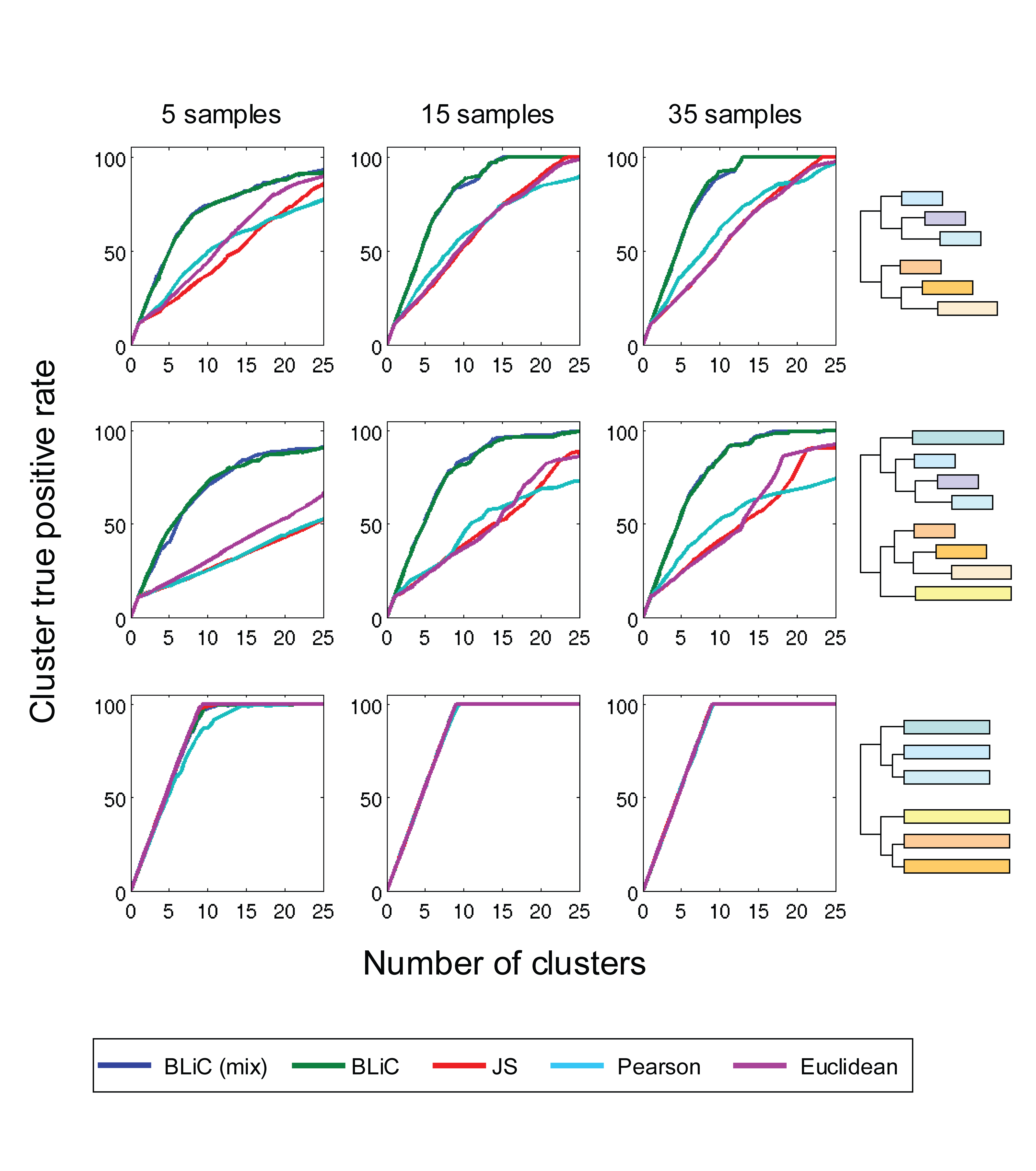

Supplement: Figure S3 — Evaluation of motif clustering. Using different subsets of motifs out of the “Yeast” data set, we compare our BLiC score (green, using a Dirichlet prior, and blue, using a Dirichlet-mixture prior) with other similarity scores: Jensen-Shannon divergence (red), Euclidean distance (purple) and Pearson Correlation coefficient (cyan). Each of the nine panels represents the average performance of 9 repeats of clustering over different motif sets. The columns correspond to the number of samples used for constructing the motifs. The rows correspond to different choices of motif sets (illustrated by the logos on the right): In the top row we cluster all motifs of partial offsets. In the middle row, we cluster all motifs, including full-length motifs and partial offsets. In the bottom row, we cluster only full motifs. In each panel we plot True Positive Rate (y-axis) vs. number of clusters (x-axis) as in Figure 4B. (1.88 MB TIF) [file pcbi.1000010.s003.tif]

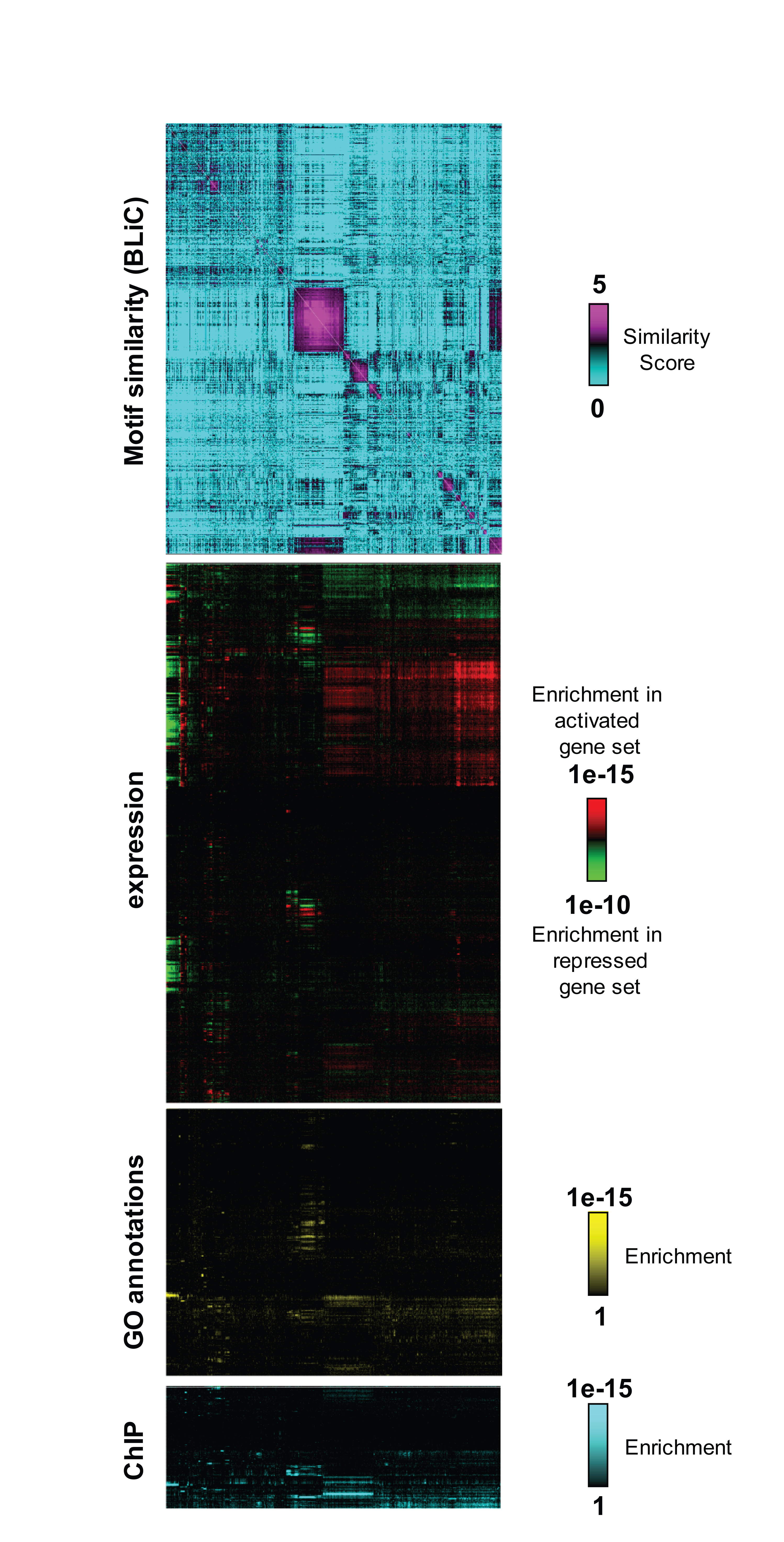

Supplement: Figure S4 — Overview of the discovered motifs. Investigation of the properties of discovered motifs. Each motif (column) is compared to other motifs using the BLiC score (rows, top group), to average expression of its targets in different experiments [30]–[32] (second group), to enrichment of its targets in GO annotations [29] (third groups) and in ChIP-chip location assays [13] (bottom groups). The rows and columns were clustered using the EdgeCluster [33] algorithm, which integrates various sources of information into the clustering process. These information sources are attributes of motifs and pairwise information about motifs. The results are clusters of motifs that have not only similar attributes, as in regular clustering algorithm, but also similar relations to motifs in other clusters. (8.43 MB TIF) [file pcbi.1000010.s004.tif]

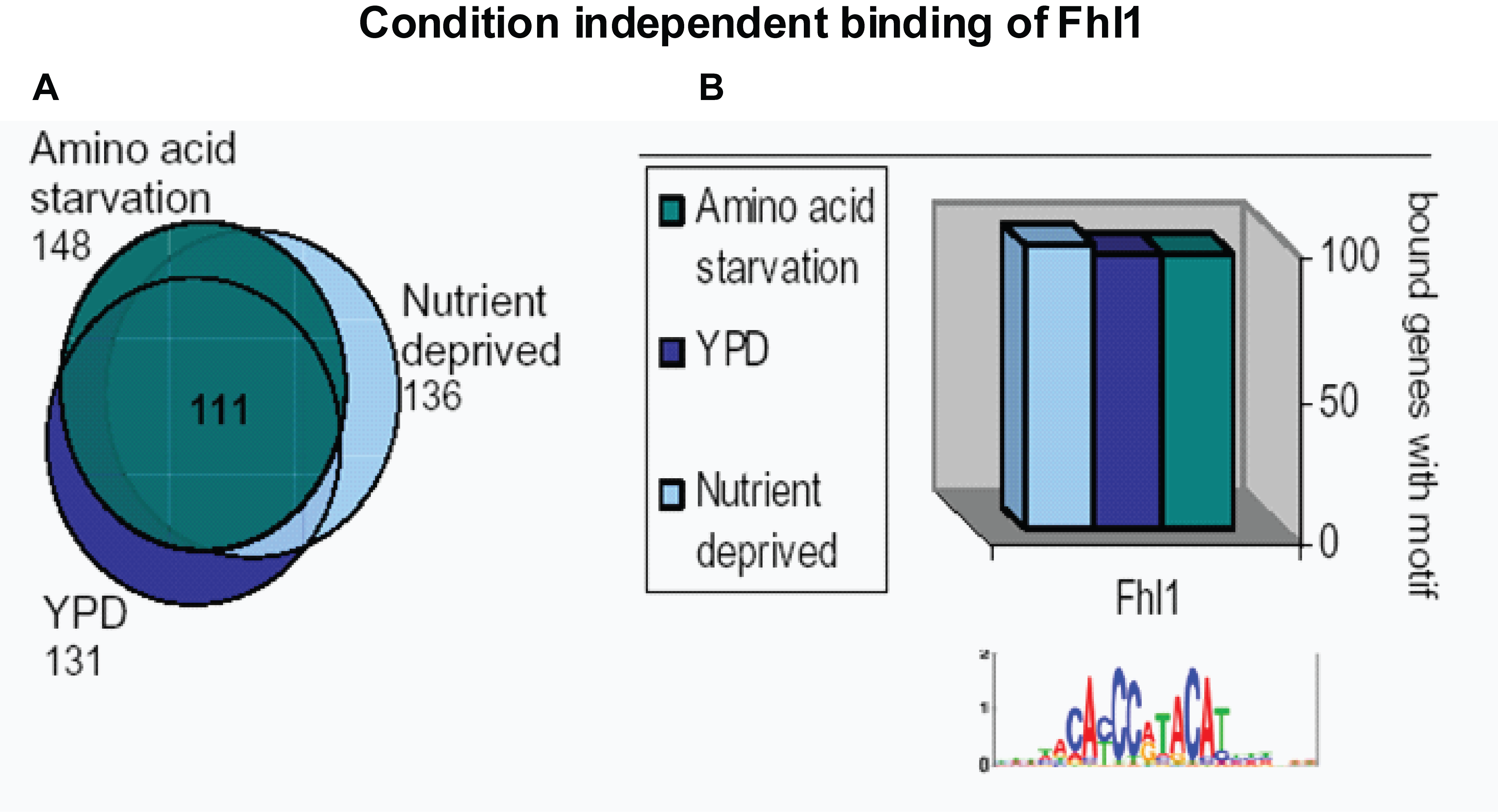

Supplement: Figure S5 — Condition independent binding of Fhl1. (A) A Venn diagram representing the results of the ChIP-chip experiment [13] for the transcription factor Fhl1 under YPD conditions, amino-acid starvation and nutrient deprived conditions. The targets of Fhl1 do not change under these three environments. (B) Under all conditions the same motif is found to be highly enriched. (2.70 MB TIF) [file pcbi.1000010.s005.tif]

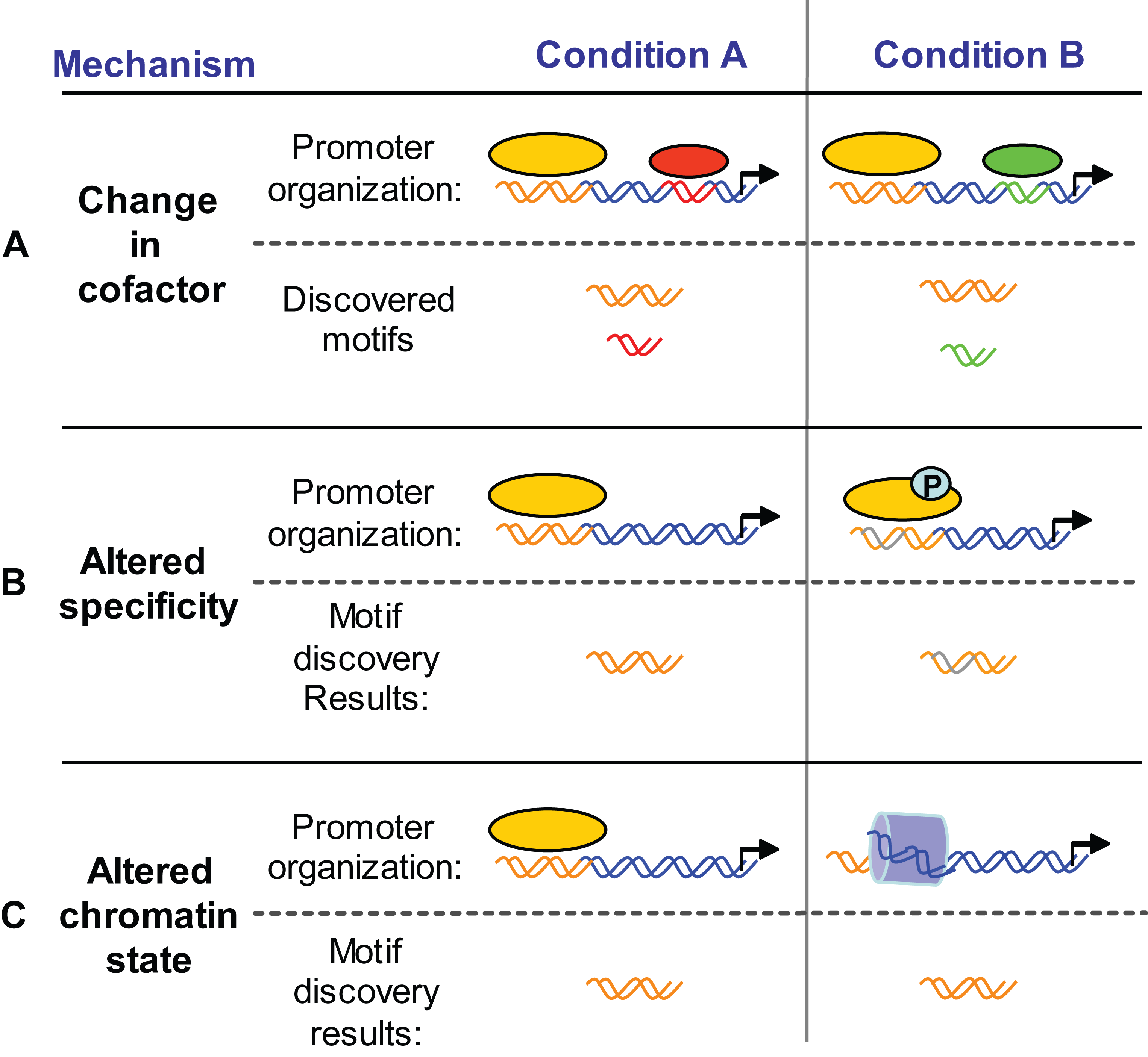

Supplement: Figure S6 — Possible mechanisms for condition-dependent binding of TFs. Motif analysis for condition-dependent transcription factors that bind different targets under different conditions. Here, three possible mechanisms that may be involved in monitoring condition-dependent binding, which lead to altered targets, are presented schematically. For each mechanism we show the scheme of the promoter organization of the target genes (above the dashed line) and the result of motif discovery (under the dashed line). (A) The first mechanism is through a change in the cofactor. This may be detected through co-occurrence of motifs of different factors. (B) The second mechanism is through a change in the specificity to the DNA. This change can be traced by identifying variations in the DNA motif. (C) The third mechanism is a change in the chromatin state. This change cannot be traced using motif analysis. (0.98 MB TIF) [file pcbi.1000010.s006.tif]
